# The Use of Genetic and Gene Technologies in Shaping Modern Rapeseed Cultivars (*Brassica napus* L.)

**DOI:** 10.3390/genes11101161

**Published:** 2020-09-30

**Authors:** Linh Bao Ton, Ting Xiang Neik, Jacqueline Batley

**Affiliations:** 1 School of Biological Science, The University of Western Australia, Perth, WA 6009, Australia; linh.ton@research.uwa.edu.au; 2 Sunway College Kuala Lumpur, No. 2, Jalan Universiti, Bandar Sunway, Selangor 47500, Malaysia; tingxiang@gmail.com

**Keywords:** canola, *Brassica napus*, genetics, gene technology, genomics, disease resistance

## Abstract

Since their domestication, Brassica oilseed species have undergone progressive transformation allied with the development of breeding and molecular technologies. The canola (*Brassica napus*) crop has rapidly expanded globally in the last 30 years with intensive innovations in canola varieties, providing for a wider range of markets apart from the food industry. The breeding efforts of *B. napus*, the main source of canola oil and canola meal, have been mainly focused on improving seed yield, oil quality, and meal quality along with disease resistance, abiotic stress tolerance, and herbicide resistance. The revolution in genetics and gene technologies, including genetic mapping, molecular markers, genomic tools, and gene technology, especially gene editing tools, has allowed an understanding of the complex genetic makeup and gene functions in the major bioprocesses of the Brassicales, especially Brassica oil crops. Here, we provide an overview on the contributions of these technologies in improving the major traits of *B. napus* and discuss their potential use to accomplish new improvement targets.

## 1. Introduction 

Rapeseed includes the Brassica oilseed crops in which *Brassica napus* is the leading crop globally, while *Brassica juncea*, *Brassica rapa*, *Brassica carinata*, and *Brassica nigra* are cultivated in selected regions of the world [1]. The term “canola” refers to the modified rapeseed low in erucic acid (<2%) and glucosinolate (<30 µmol/g of dried defatted meal) content, which includes cultivars of *B. napus*, *B. rapa*, and *B. juncea* [2]. The allopolyploid oilseed plants were formed following interspecific hybridization events, for example, *B. napus* (AACC, 2n = 38) was formed by hybridization of *B. oleracea* (CC, 2n = 18) and *B. rapa* (AA, 2n = 20) ~7500 years ago [3]. Modern approaches in evolutionary analysis have attempted to consolidate the origin of *B. napus* [4,5]. The use of rapeseed oil in lamps has been recorded since 2000 BC in India and since the 13th century in Europe [2] with additional uses in making food and soap [6]. Rapeseed cultivars were introduced to Canada in 1936 [7] for lubricant production for war ships, and was first commercially grown in Australia in 1969 [2].

Intensive breeding programs in *B. napus* were initiated in many countries around 1970, including Australia [2]. Back-crossing with the low-erucic- acid line, Liho, brought successful release of the first low erucic-acid cultivars of *B. napus* and *B. rapa* in 1968 and 1971, respectively [7]. High oleic and low linolenic (HOLL, > 65% oleic acid and < 3% linolenic acid) rapeseed genotypes were created by chemical mutagenesis using ethyl methanesulfonate (EMS) [8,9], and HOLL varieties have been commercially grown in Canada since 2005 and in Australia since 2006 [10]. The emergence of genomic and genetic tools has facilitated breeding programs [11,12] leading to the release of multiple rapeseed/canola varieties such as high erucic acid rapeseed (HEAR) [13], for non-food purposes, and varieties for food industries e.g., high lauric acid canola Laurical™, Roundup Ready®(Monsanto, Missouri, US), InVigorTM (BASF), with provisional herbicide tolerance (HT) and resistance against major pathogens. The parental lines for rapeseed breeding programs varied depending on the geographic location, with the progenitors contributing towards the three ecotypes of *B. napus*, spring type which is widely cultivated in Canada, Australia, and northern Europe, winter type *B. napus* which is predominant in Asia and the remaining area of Europe [14], and semi-winter type as the primary rapeseed in China [15]. The increasing human population and sustainable energy initiatives have led to a high demand of canola oil for the food industry, biofuel, and various industrial purposes [16], prompting investments for further improvement of *B. napus*, with emphasis on the major traits relating to oil yield and quality, meal quality, herbicide tolerance, biotic and abiotic stress tolerance [17,18]. Fast-pace development of genetic and gene technologies has facilitated the identification of those breeding targets as well as achieving significant advances in the improvement of canola genomics resources. 

During the last 20 years, dramatic innovations in next-generation sequencing (NGS) technologies have led to a significant drop in sequencing cost and offered exciting opportunities to explore plant genomes [19]. Recent rapeseed genomes were assembled using Illumina high-throughput short read sequence technologies and third generation sequencing technologies, notably, single molecule real-time (SMRT) sequencing (PacBio) and Nanopore sequencing technologies (Oxford Nanopore Technologies, Oxford, UK) capable of generating long-read sequences [20,21]. Genome assembly in highly complex genomic regions frequently found in *Brassica* species can be greatly improved using optical mapping [22,23] and chromosome conformation capture (Hi-C) technologies [23]. 

High-throughput SNP marker identification and genotyping of *Brassica* species, achieved through NGS, allow efficient identification of quantitative trait loci (QTL) and generation of high density genetic maps, which means there is a higher chance of detection of a candidate gene linked to a nearby SNP [24,25]. The SNP markers are useful for genome-wide association studies (GWAS), marker-assisted selection (MAS), genomic selection (GS), and germplasm identification [26]. For example, SNP markers have been widely deployed in mapping QTL conferring resistance to blackleg, Sclerotinia stem rot [27] and clubroot [28] in *Brassica*. In addition, genotyping can be implemented quickly using the *Brassica* 60K Illumina Infinium™ array with the capability to genotype of 52,157 SNPs in *B. napus* [29]. High-throughput genotyping technologies allow large-scale genomic characterisation of the huge *Brassica* germplasm collections worldwide comprising > 74,000 *Brassica* accessions mainly stored in The Netherlands, Norway, Spain, UK, US, and Australia [30] to facilitate improvement and broaden the genetic diversity of *Brassica* varieties.

Limitations in gene pool diversity [31,32], and the time and laborious constraints in canola breeding can now be overcome by using genetic modification [32] and genome editing technologies whereby transgenes or mutations can be directly introduced into plants, providing additional ways to investigate gene functions in biological processes [33,34,35,36]. The most commonly applied genome editing tool in eukaryotes in the last 8 years, the clustered regularly interspaced short palindromic repeat (CRISPR)/CRISPR-associated protein (Cas) system [37,38], is gaining more interest compared to conventional genetic modification methods due to its high efficiency in targeted nucleotide modification and generation of a transgene-free end-product [39]. In a CRISPR/Cas system, a single-guide RNA (sgRNA) binds to the Cas protein and directs it to one or more specific target sequences [40] marked by a short sequence, protospacer-adjacent motif (PAM), located at the 3’ downstream position [37] and varying in accordance with the type of Cas enzyme utilized [40,41]. Recently, a platform for induction of mutagenesis in oilseed rape via the CRISPR/Cas system has been developed [42], facilitating early utilization of this tool in improving agronomic traits of *B. napus* e.g., plant height [43], silique development [44], pod shatter resistance [45], and flowering time [46]. This genome editing tool provides a faster route to interpret the relationship between genes and phenotypic traits controlled by complex genetic structure, biosynthetic pathways, and regulatory elements [26].

Development of breeding techniques and the genetics and genomics associated technologies described above have improved the quality and agronomic traits of canola varieties, which make the current canola varieties well-suited for expectations of growers, processing sectors, and final users [47,48]. The advent of technologies, including omics technologies, genetic transformation, and genome editing, have facilitated deeper understanding of the physiological and biochemical processes regulating the development of the major phenotypic traits of *Brassica* oilseed crops, which has brought tremendous improvements in the desirable traits of canola in the last decade [49,50]. This review provides a summary of their impressive contributions towards improvement and innovations of canola varieties. 

## 2. Improvement and Innovations of Canola Varieties

### 2.1. Higher Resolution for Rapeseed Genome Characterization 

Genomic data is an integral part in developing breeding strategies and exploiting genetic potential of germplasm. The revolution in sequencing technologies has accelerated genome research, where it used to be an obstacle for polyploid species [21]. Using a combination of NGS and Sanger sequencing technologies, the first genome of *B. napus* was sequenced, from which its evolutionary history was clarified with the linkages to its ancient ancestor [5]. Meanwhile, the combination of PacBio’s SMRT and NGS short read sequencing technologies have been deployed to study the various forms and complexity of the gene transcripts responsible for another development in *B. rapa* (Chinese cabbage), providing comprehensive transcriptome data for better accuracy of genome annotation [51]. Version 3 of the *B. rapa* genome was assembled using combinations of SMRT, optical mapping (BioNano), and Hi-C technologies with up ~ 30-fold improvement compared to the previous versions [52]. Other more recent genome assemblies for the *Brassica* species are *B. olerecea* accessions JZS v2 [53], *B. nigra* [54], *B. napus* ‘Darmor-bzh [55], and German winter *B. napus* Express 617 [56]. These *Brassica* reference genomes were sequenced and assembled long-read sequencing technologies using SMRT and Oxford Nanopore Technologies (MinION and higher throughput version of the MinION, PromethION) complemented with Illumina data and verified by optical mapping and/or Hi-C data [52,56,57,58]. Qiao et al. [59] have proven cytoplasmic genomes from mitochondria and cytoplasms would be a highly effective approach for phylogenetic and evolutionary studies on *B. napus* including employing this resource in improved breeding. 

### 2.2. Pan-Genome

Although there is an increasing amount of reference genome data available for *Brassica* species, a high-quality reference genome comprised of genome data of individuals within a species is mandatory for comparison studies and evaluation of genetic diversity, which is essential for improvement of breeding [57]. The original effort in assembling a core genome representing a population of *Streptoccocus agalactiae* [60] introduced the new term “Pan-genome”. Pan-genomics exploits the genomic content of all the individual lines of a species under study, including closely related species such as crop wild relatives, differentiating the core genes which are genes present in every individual of the species, from variable genes, which are genes present only in one or some individuals [61,62]. The variations in the genomes can be in many forms including SNP, copy number variation, and presence-absence variation (PAV). These may arise due to many rounds of DNA exchange events happening within or between the (sub)genomes, accumulated within a species during the evolution from the ancestral species and subsequent breeding history with different genetic backgrounds, resulting in high species diversity [63]. The first pan-genome of *B. rapa* was constructed by comparing genomic data of *B. rapa* species including Chinese cabbage cultivar, Chiifu; an oil-like rapid cycling line (RC-144) and a Japanese vegetable turnip [64], which is 283.84 Mb in size and includes 41,052 predicted gene models, of which 90% are the common genes and 9% are unique genes only found in one of the three genomes. Constructed by an iterative assembly approach, the pan-genome of *B. oleracea* revealed the number of variable genes was roughly more than 20% of the total pan-genome gene number; 12,806 (out of 61,379 pan-genome genes) [65], whilst in *B. napus* over one third of the genes were variable: 35,481 (out of the total 94,013 genes in the pan genome) [66]. Recently, by using a de novo approach, a pan-genome representing 9 *B. napus* genomes was obtained comprising of 58,714 (~56%) core-gene clusters present in at least seven genomes, 44,035 (~42%) dispensable gene clusters and around 2% specific genes [57]. 

Further dissection of gene functions within pan-genomes across species showed dispensable genes may have higher mutation rates, and are less functionally conserved compared to core genes, of which functions are associated with responsive mechanisms to biotic and abiotic signals [62]. In the pan-genome of *B. oleracea*, Bayer et al. [67] found that the most abundant resistance gene analog (RGA) candidates in the additional pan-genome contigs were leucine rich repeat (NBS-LRR) genes compared to receptor-like kinase (RLK) and receptor-like protein (RLP) genes, and identified 59 RGA candidates linked to known QTL of *Sclerotinia*, clubroot, and *Fusarium* resistance. It was found that 753 out of the total 1749 RGAs in the pan-genome of *B. napus* [68] are variable, 368 of which are not present in the reference genome (cv. Darmor-bzh), suggesting many genes relating to plant resistance mechanisms may be unknown. This is consistent with the fact that the core genes (10584 SNPs identified, 70.97%) contained more SNPs than variable genes (4734 SNPs identified in 299 genes), and 688 SNPs were found in 106 RGAs within QTL (*LepR1*, *LepR2*, *Rlm1*, *Rlm3*, *Rlm4*, *Rlm7*, and *Rlm9*) conferring blackleg resistance in *B. napus* [68]. Transcriptomic analysis coupled with pathotyping could assist functional annotation of RGA candidates [69,70] while their roles in responsive mechanisms could be validated using gene technology or/and CRISPR/Cas tool [71]. Pan-genomes of *Brassica* oilseed crop provide insights on the genetic variations leading to phenotypic variations, differential trait expression of individuals within species which supports finding new candidate genes. Higher coverage of pangenomes allows more precise characterization and prediction of gene content of individuals within a species [63]. 

## 3. Breeding for Economically Important Agronomic Traits of *B. napus*

### 3.1. Oil Content and Specialty as Priority

Canola oil, the main product of *B. napus*, is the most consumed vegetable oil worldwide [72]. Canola food oil possesses a unique fatty acid profile compared to other types of vegetable oil: low in saturated fatty acid (SFAs), typically 7%; high monounsaturated fatty acids (MUFAs) and polyunsaturated fatty acid (PUFAs), comprised of 61% oleic acid, 21% linoleic acid, and 11% alpha-linolenic acid (ALA) [73]. Phytosterols and vitamin E are additional healthy components of canola oil [74]. The health benefits of a canola oil-based diet were clinically proven as reduction of blood glucose levels and risks of coronary heart disease, promoting immunity and prevention of tumor cell growth, contributed by MUFAs, PUFAs, and vitamin E [75]. For cooking purposes and biofuel production, canola oil with high oleic acid (C18:1) and low linolenic acid (C18:3) is preferred through providing high stability and longer shelf-life for the oil [12,76].

**Figure 1:**
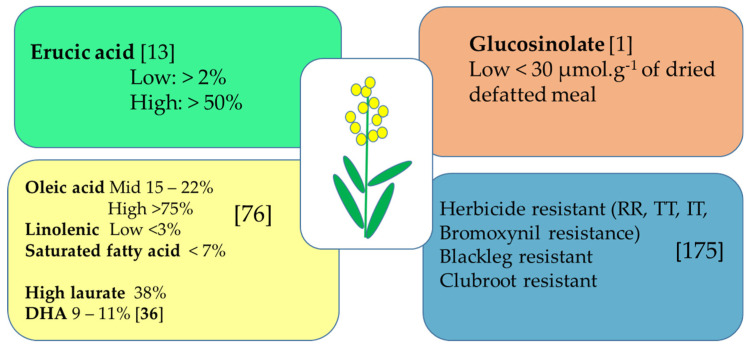
Desired optimal traits in current canola varieties, specifically for oil and meal content, herbicide, and disease resistance.

There are tailored profiles of canola fatty acids required for specified industries and industrial applications which were achieved through conventional breeding and mutagenesis during the 1990s [76], Figure 1. To meet the demands of the increasing market of cooking oil, priority for improvement of canola varieties is an increase in oleic acid (C18:1 or ω-9) level and decrease in linolenic acid (C18: 3 or ω-3) and SFAs with specific targets varying by country. For example, in Australia, the aim is for up to 67–75% for oleic acid and less than 3% linolenic acid [77]. Expansion of the acreage of high oleic and specialty canola varieties (to 33% of canola acres) is also part of Canada’s scope for the canola crop in the period 2015–2025, as well as further reducing SFAs to below 6.8%, and especially palmitic and stearic levels below 4% [76,78]. To maximize canola intake in both biofuel and feed industries, varieties with high oleic acid and extremely low glucosinolate content are targeted [12]. High oil yield (40–45% of the mass) and low production cost are features making canola a potential crop for producing high demand fatty acids [36]. 

Understanding biosynthesis of the major C18 unsaturated fatty acids (UFA) in prokaryotes and eukaryotes has been well documented since the 1990s, where synthesis pathways, key fatty acid synthetic enzymes, and key regulating factors have been identified [79]. In *B. napus*, fatty acid desaturase (*FAD*) genes, specifically *FAD2* and *FAD3*, together with stearoyl-acyl carrier protein desaturase (*SAD*) catalyzing desaturation of steric acid (C18:0) to C18:1 are a focus for controlling oil quality [79], however they were also found to be involved in abiotic stress tolerance such as high cadmium (250 µM) and salinity (100 mM) conditions [80]. Elevated oleic levels were reported in lines with mutated *FAD2* genes [35,81]. Targeted mutagenesis of a *FAD2-Aa* allele of *B. napus* using CRISPR/Cas9 produced the heritable mutant, *fad2_Aa* allele with a 4-bp deletion, which was separated from the transgenes by backcrossing [35]. By genome wide analysis of the *FAD* gene family, 84, 45, and 44 *FAD* genes were identified in *B. napus*, *B. rapa*, and *B. oleracea* genomes, respectively, with different distribution, which were assigned into four and six subfamilies in term of soluble and membrane-bound *FAD*s, respectively [82]. Based on 201,187 SNP markers developed from SLAFseq (specific locus amplified fragment sequencing) and GWAS of four important fatty acid content traits (erucic acid, oleic acid, linoleic acid, and linolenic acid), 148 SNP loci were found significantly associated with these traits and 20 orthologs of the candidate genes regulating the fatty acid biosynthesis (fatty acid synthesis, desaturation, elongation, and metabolism) and 14 candidate genes on chromosomes A8 and C3 with potential contributions were identified [83]. Roles of *BnTT8* genes in *B. napus*, the conserved gene complex controlling flavonoid accumulation in plant crops, have been elucidated by using the CRISPR/Cas9 tool to induce targeted mutations at *BnA09.TT8* and *BnC09.TT8b* genes, in which the mutant lines produced seeds with a yellow seed coat, significant increase in seed oil and protein content, and altered FA composition [84]. 

High demand long chain-unsaturated fatty acids, such as eicosapentaenoic acid (EPA) and docosahexaenoic acid (DHA), which were once exclusively obtained from fish, are now novel components of transgenic *B. napus* lines [36,85]. In 2005, by transformation using *A. tumefaciens* containing 6 different constructs of desaturase genes from *Thraustochytrium* sp., *Pythium irregular* and *Calendula officinalis*, and fatty acid elongase from *Physcomitrella patens* in *B. juncea*, very long chain PUFAs such as arachidonic acid (AA) and EPA were produced at levels of up to 25% and 15% of total seed fatty acids, however, the DHA biosynthetic pathway needed further optimization [34]. Using a similar approach, Walsh et al. [85] produced transgenic *B. napus* expressing PUFA synthases (*OrfA*, *OrfB*, and hybrid *OrfC*) from *Schizochytrium* sp. ATCC 20888 (Schizo20888). These microalgal PUFA synthases assemble C2 units from malonyl-CoA into long chain PUFAs in cytoplasm with local NADPH supplement. The average DHA contents in T2 seeds from the inbred lines of the selected events were around 2.87–3.43%, and the total content of both DHA and EPA was around 4.4% in field-produced canola oil. In the latest transgenic DHA canola variety by Petrie et al. [36], seven fatty acid biosynthesis genes from yeast and microalgae were de novo synthesized as a single fragment of 19,750 bp and regulate DHA production through the delta-6-desaturase aerobic long-chain (C20) polyunsaturated fatty acid synthesis pathways in *B. napus*. Levels of DHA from 9 to 11%, similar to those obtained from fish, were obtained from the best transformation event (NS-B50027-4) in open field trials in Australia and Canada. With the fact that the DHA canola is approved for cultivation for human and animal consumption of the oil in Australia and cultivation approval in the US [86,87], production of other valuable fatty acids through genetic engineering tools is quite promising. 

Regarding renewable and sustainable resources, erucic acid from canola oil is an excellent raw material in producing polymers used in film manufacture, nylon, lubricant, and emolient industries [13,17]. Furthermore, with the increasing demand of canola oil for food and efforts to promote renewable resources of high erucic acid rapeseed (HEAR, 45–60% erucic acid), varieties have been grown in European countries for biofuel and raw oil supplement (<2% of total weight of crushed seeds) for human consumption [88,89]. By overexpressing the fatty acid elongase gene (*fae1*) simultaneously with the lysophosphatidic acid acyltransferase gene from *Limnanthes douglasii* (Ld-LPAAT), erucic acid synthesis was predominant over PUFA synthesis in competitive elongation to the triaclyglycerol backbone in transgenic lines, which resulted in an increase in erucic acid content of up to 72% and a PUFA content as low as 6% [13]. The roles of LPAAT genes of *B. napus*, BnLPAT2 and BnLPAT5, in regulating oil biosynthesis has been recently confirmed by targeted mutations using Cas9 with single-gRNAs and multi-sgRNAs whereby the resulted *Bnlat2* and *Bnlat5* mutants showed decreased oil content and enlarged oil bodies [33]. Cytoplasmic genomes are also attracting more interest as the new approach for maximizing oil content in canola [90].

### 3.2. Exploiting Canola Meal Potential 

Despite the protein-rich content of canola meal (38%, by meal weight) and wide adoption of low glucosinolate (GSL) varieties, antinutritional compounds, typically, phenolics, sinapine, and phytate, are still challenging the use of by-products of canola oil production as animal feed [16,91]. The expansion of biofuel production from canola [89] has led to significantly additional volumes of canola meal as a by-product and promoted feed industry [16,92]. However, in the study of Skugor et al. [93], the replacement of rapeseed meal for soybean meal as 20% supplement of diet for growing finishing pigs in 3 months, produced elevated expression of major control factors of energy generation, reduced protein synthesis in muscle tissue, and greater expression of oxidative stress, which was caused by higher fiber content and GSL compounds and other secondary metabolites in rapeseed meal. Other studies on animal nutrition also had similar conclusions about antinutritional compounds in canola meal, including high fiber content, lignin, and GSLs [94,95].

Since the release of the first “double-low” canola cultivar with low levels of both erucic acid and glucosinolates (GSL), breeding efforts to further reduce GSL content in canola cultivars including *B. rapa*, *B. napus*, and *B. carinata* are still in progress [1]. While GSL inclusion in diet affects health of livestock e.g., damaging liver, kidney, and thyroid gland and impairing fertility [96], this secondary metabolite could be useful as a cancer prevention and plant protection agent [97,98]. The importance of GSLs in plant defense has been demonstrated [99] and explains the increase in susceptibility to pests and diseases of the “00” (low seed erucic acid and GSL) rapeseed cultivars [100]. 

Over the at least 15 years, knowledge on metabolism of GSLs in Brassica crops and Arabidopsis, and the underlying genetic architecture has been achieved [97,98,100,101]. To date, there are at least 130 different classes of plant GSL, almost all from the Brassicales order, summarized by Blažević et al. [102] and approximately 40 different GSLs synthesized in Arabidopsis [103]. Understanding the regulation of GSL biosynthesis through molecular genetic tools (SNP marker identification, QTL mapping) and gene technology (gene cloning, gene knock-out) enables controlling in planta GSL profiles and their targeted position for expression [100]. Using an associative Transcriptomics platform comprising 355,536 SNPs and a transcriptome reference, Kittipol et al. [100] found a region on chromosome A3 of *B. napus* had strong association with variation in root aromatic GSL and confirmed the gene *Bna*.HAG3.A3 as a key regulator for the trait expression. Although studies of genetic factors controlling expression of GSL in vegetative parts and seeds of canola are being performed, they are clearly the keys to enhance pest resistance for canola varieties, and increase nutrition value and use of rapeseed meal. Future canola varieties with differential expression of GSLs in term of types, levels, and position in plant from which the obtained meal may increase in its intake for the feed industry and expand to other markets (as in cancer treatment, herbicide industry). 

Among efforts to accommodate higher-value markets for canola meal, breeding towards enhancement of canola protein content is the most achievable and economical solution [16,104]. Canola protein extracted from the meal with a well-balanced amino acid profile could be a new protein source for humans, providing that allergen causing proteins, napin and cruciferin, are controlled along with antinutritional compounds residing in the seed hull [104]. Therefore, improvement targets for protein from canola meal should be protein content, protein types (napin, cruciferin), and reduction in fiber and GSL [104]. To recognize this, recently Canadian Protein Industries announced a major investment in breeding to improve protein and reduce fiber to diversify markets for food and feed applications of canola meal [105]. 

Major genetic factors controlling seed cellulose are being investigated in *B. napus* and the model plant *Arabidopsis* [106], providing fundamental knowledge about the genetic control of fiber and protein content of seed. Using RNA interference technology, the functions of the two genes encoding cellulose synthase subunits in *Arabidopsis thaliana*; AtCESA1 and AtCESA9, were elucidated by generating transgenic *A. thaliana* containing the hairpin RNA (hpRNAi) constructs which specifically spliced their complementary targets [106].

Yellow-seed rapeseed varieties of *B. napus*, *B. rapa*, and *B. juncea* have been shown to have lower fiber content than brown-seeded canola, thus, showed higher feed efficiency in an in vivo experiment on chickens than brown-seed canola [94]. The characteristic genetic background of yellow-seeded *B. napus* could be differentiated from the black-seeded ones by comparing the correlation between differentially expressed genes (DEGs) from the transcriptomic data and the observed traits in the two *B. napus* types, from which down-regulated DEGs in the yellow seeds were linked to reduction in flavonoid and lignin contents, while other DEGs linked to fatty acid biosynthesis and metabolism [107]. Post-translation regulation of seed coat traits could be an effective approach with less effect on cellulose biosynthesis in other plant parts [106]. There have been at least 2 examples of development of yellow seeded line; through directed mutation, an improved yellow-seeded rapeseed line was produced [108]. In addition, in 2007, a new *B. napus* line was introduced by Canadian scientists with low fiber, yellow canola seeds comprising high oleic, low linolenic oil [109]. However, yellow-seed canola is still yet to be commercially successful. 

### 3.3. Disease Resistance

Common diseases in rapeseed reported worldwide includes blackleg disease, caused by *Leptosphaeria maculans* [110,111] clubroot, caused by *Plasmodiopora brassicae* [112,113] and Sclerotinia stem rot, caused by *Sclerotinia sclerotiorum* [114,115]. The availability of genomic data and genetic maps of *Brassica* species, along with associated bioinformatics tools, allow deeper dissection of the genetic architecture of resistance. An efficient platform for identification of disease resistance genes can be implemented based on resistance gene analogs (RGAs) with the use of pangenomics data and confirmed SNP markers [68]. Taking this approach, Dolatabadian et al. [68] identified a total of 15,318 SNPs associated within 1030 *R*-genes in the *B. napus* pan-genome, which facilitates identification of candidate blackleg *R*-genes. Focusing on *Brassica* improvement for biotic stress, a study on pan-genomics in *B. oleracea* showed that the wild C genome species, *B. macrocarpa* contains the highest number of disease resistance gene candidates (1495) compared to the average RGAs of the cultivars (1450) [67], inferring that the crop wild relatives have high gene diversity with novel alleles which are suitable for introgression into cultivated *Brassica* species to improve varieties of *B. napus*. 

#### 3.3.1. Blackleg Resistance

Blackleg disease, caused by *Leptosphaeria maculans,* is still the major constraint for the main canola producers including Australia, Canada, Europe, and the United States, despite blackleg resistant cultivars being adopted roughly 30 years ago and undergoing continuous improvement [27,110,111,116,117,118,119]. The host-pathogen interaction during the disease process has been clearly understood based on pathotyping and molecular techniques (PCR, nucleotide sequencing, and gene technology) in which qualitative (major genes) and quantitative (multiple genes) resistance is triggered [120,121]. Molecular and gene technologies allied with fast development of bioinformatics has accelerated the identifications of resistance genes (*R*) in the host plants and avirulence genes (*Avr*) in *L. maculans*. At least 19 major *R* genes against *L. maculans* have been genetically mapped in *B. juncea*, *B. rapa*, and *B. napus* [119,120], with 3 genes cloned [120,122]. By contrast, nine of the 16 *Avr* genes identified in *L. maculans* have been cloned to date [123]. Reliance on a single *R* gene and little or no rotation with less susceptible crops leads to rapid evolution of *L. maculans* populations and resistance breakdown [119,120]. Using a GWAS approach, Fu et al. [116] identified 32 and 13 SNPs from Canada and Chinese accessions tightly linked with blackleg resistance which were distributed on chromosomes A03, A05, A08, A09, C01, C04, C05, and C07, in which potential SNPs located on A8 associated with resistance to 12 *L. maculans* isolates and 25 RGAs were identified within this region. Analysis of quantitative resistance (QR) to *L. maculans* in 177 double haploid lines under three experimental conditions using DArTseq markers revealed 3 SNP associations for QR on chromosomes A3, A4, and A7 [117]. New strategies for identification of genes relating to blackleg resistance is quite promising as providing an efficient way to obtain and evaluate new *R* genes in accordance with race structure of *L. maculans*, which is crucial for development of new rapeseed varieties [110,111].

#### 3.3.2. Clubroot Resistance

Clubroot, caused by the soil-borne biotrophic pathogen *Plasmodiopora brassicae*, has long been an important canola disease and has recently been increasing in Europe, China, India, Nepal, and Australia [124,125,126,127,128,129]. The yield losses by this disease can be around 60–90% when susceptible canola/rapeseed cultivars are planted in *P. brassicae* infested field [129]. Chemical treatment or sanitation practices for eradication of the pathogen from infested cultivating soil is impractical and impossible [113], which suggests utilization of resistant cultivars is the most effective and sustainable solution [125]. Clubroot resistance (CR) genes and gene loci contributing to the resistance in Brassica oilseed crops have been identified and mapped. Clubroot resistance has been discovered to be controlled by dominant loci in *B. rapa*, with two resistant genes being cloned, [130,131], 13 CR loci mapped on chromosomes A01, A02, A03, A06, A08 and the most recently A05 with CrrA5 [125,132,133]. At least 20 QTL in the C genome of *B. oleracea* [28,134] and more than 30 QTL in the A C genome of *B. napus* conferring CR have also been identified and mapped [134]. The CR gene (*Rcr6*) on chromosome B3 of *B. nigra* (BB, n = 8), was the first CR gene identified and mapped in the B-genome of Brassica species [132]. Utilization of modern genomic approaches e.g., GBS (genotyping by sequencing), GWAS, and RNA sequence analysis has facilitated identification of a number of CR candidate genes corresponding to specific race/pathotype of *P. brassicae* in *B. oleracea*, *B. rapa*, *B. napus*, which were summarized by Zhou et al. [135]. These valuable CR resources need to be further explored and precisely identified by molecular markers for breeding CR and dealing with resistance overcome by novel strains of *P. brassicae* [28,134,136]. By mapping RNA sequences of *B. napus* genotypes with different response to *P. brassicae* pathotype 5X to the *B. napus* reference genome (AST_PRJEB5043_v1), Galindo-González et al. [70] found differential expression in these genotypes whereby the standard defense-related proteins e.g., chitinases and thaumatins, and salicylic acid-mediated responses were up regulated in the resistant genotype coupled with mostly inhibited jasmonic acid-mediated responses. The study also identified major proteins relating to *P. brassicae* resistance and susceptibility e.g., calmodulin binding protein 60g (CBP60g), systemic-acquired resistant deficient 1 (SARD1), and xyloglucan endotransglucosylase/hydrolase (XTH).

#### 3.3.3. *Sclerotinia* Resistance

*Sclerotinia sclerotioum* is a devastating fungal pathogen infecting more than 400 plant species across 75 families [137]. The typical symptoms caused by *Sclerotinia* pathogens include water-soaked rotting of leaves, stems, or fruit covered by fluffy fungal mycelium which subsequently develop compact resting bodies or sclerotia [115]. For over 15 years, its interaction with host plants has been investigated at the molecular level due to the complication of *S. sclerotiorum* possessing both biotrophic and necrotrophic lifestyles, which involves a variety biological processes including reactive oxygen species, lipid and calcium signaling, and DNA methylation-mediated transcription regulation [31]. This diverse interaction may be caused by a large set of candidate effector proteins, which can be identified through bioinformatic research [138]. Efforts in producing *Sclerotinia*-resistant canola varieties includes mutagenesis [139,140], breeding resistance [141,142], and genetic manipulation [143]. In 2011, a *Sclerotinia*-resistant *B. napus* variety was released by Pioneer Hi Bred International [144], however, there is limited information about quantitative disease resistance to the pathogen and associated QTL. By integrating QTL for the resistance, 26 candidate NBS-LRR genes in *B. napus* were found associated with *S. slerotiorum* resistance [145]. Through a transcriptome analysis approach, Chittem et al. [69] confirmed major pathways underlying communications between *S. sclerotiorum* and *B. napus* and the associated key genes. Based on knowledge on the plant immunity involvement of WRKY transcription factors in *Arabidopsis thaliana*, the roles of the corresponding WRKY genes in *B. napus*, *BnWRKY11*, and *BnWRKY70*, were elucidated using Cas9 enzymes and sgRNAs to induce nucleotide specific mutations in these genes, and the results showed the increased resistance to *S. sclerotiorum* in the mutant lines of *BnWRKY70*, while *Sclerotinia* susceptible phenotype was observed in lines with overexpressed *BnWRKY70* [71]. 

### 3.4. Abiotic Stress Tolerance

*Brassica* tolerance towards key abiotic stresses such as heat, drought, cold, and salinity are critical for *Brassica* breeding programs. The physiological changes of canola and the signaling pathways involved in response to the various abiotic responses are often interconnected across the various abiotic stress types [146]. For example, the stress response genes such as the homeodomain-leucine zipper subfamily transcription factors play a role in drought tolerance of *B. napus* [147] and other abiotic stresses, supported by the findings of 113 homeobox genes being identified in Chinese cabbage as differentially expressed under multiple stresses such as cold and osmotic stress [148]. Other examples of genes related to multiple abiotic stresses include the membrane-bound FAD genes in canola that also play a role in improving oil quality. The FAD genes were found to be differentially expressed under cadmium and salinity stresses using qRT-PCR [80]. The FAD candidate genes identified from Xu et al. [80] are highly promising for breeding cadmium and salt tolerant canola. In addition, phytohormones such as abscisic acid (ABA) are commonly involved in abiotic stress response besides regulating plant growth and development [149]. Targeting the coding and transcription factor genes involved in the ABA-mediated signaling pathway and using these genes for creating transgenics *B. napus* that are tolerant towards drought, cold, osmotic, and salinity stresses is very promising [150]. Regulatory pathways of abiotic stress responses in *B. napus*, typically drought, salinity, extreme temperature, and cadmium toxicity, along with the gene families identified in *B. napus* were reviewed in detail by Lohani et al. [146] which highlighted multigenic engineering approach in coping with multiple abiotic stresses and CRISPR/Cas9 as a versatile tool to achieve this target. Mutant *B. napus* lines of paralogous genes of *Bna.RGA* family, orthologues of Arabidopsis REPRESSOR OF GA1-3 (RGA) genes functioning as repressor in gibberellin signaling, were generated via CRISPR/Cas9 with high efficiency [151] in which *bnaa6.rga-D* (gain-of-function mutant) presented enhanced drought tolerance and its stomata were hypersensitive to abscisic acid (ABA) signal, while *bnarga* (loss-of-function mutant) possessed reduced drought tolerance and less sensitivity to ABA treatment [152]. Using the CRISPR/Cas9 tool will efficiently exploit and elucidate roles of identified major genes/factors regulating stress responses in *B. napus*, providing qualified germplasm for breeding of abiotic stress tolerance. 

### 3.5. Herbicide Tolerance/Resistance 

Weed invasion is ubiquitous and a persistent threat to crop yield, especially to major field crops including canola [153], for which chemical treatment is the most effective control and constitutes considerable expenditure, approximately 20–36% of total operating cost of canola production [154,155]. Use of herbicide tolerance (HT) crops in combination with a broad spectrum herbicide as glyphosate has been assumed as revolutionary in weed management [156,157] as evidenced by their benefits to agronomy, economics, and the environment [158,159], providing flexibility in scheduling weed treatment and crop yield enhancement [160]. 

HT canola varieties have been obtained either through conventional breeding e.g., Clearfield^®^ and triazine-tolerant (TT) canola varieties, or genetic engineering e.g., InVigor^®^ (Bayer CropScience, Leverkusen, Germany) and Roundup Ready (RR, Monsanto) [158]. The first TT canola variety, developed through the incorporation of the TT trait from *B. campetris* and *B. rapa* by backcrossing [161], was quickly adopted in Canada and Australia in the 1990s. In 1995, the first imidazolinone-tolerant *B. napus* was registered, of which the trait was developed through mutagenesis by BASF, and the first transgenic canola variety tolerant to the herbicide glyphosate (Roundup) was developed by Monsanto [2]. Glyphosate tolerance in the initial RR cultivars was conferred by the expression of CP4 5-enolpyruvylshikimate-3-phosphate (EPSP) synthase from *Agrobacterium* sp. strain CP4, the enzyme involved in the shikimate pathway essential for the biosynthesis of aromatic amino acids [162]. This is the most common genetically modified (GM) trait in the current biotech crops, with over 80 transformation events being approved for commercial release [163]. Roundup Ready canola has gained approvals for commercial release in major canola producing countries including Canada, Japan, USA, and Australia within 1994 and 2003, where the obtained oil has been approved for food use in addition to European countries, Mexico, Philippines, and Singapore [164]. At least 34 transformation events have been approved in canola varieties to produce the crops with glyphosate HT alone or stacked with other GM traits [163]. Since their commercial release, HT canola varieties have dominated in canola growing countries [165]. However, the intensive cultivation of HT canola and overuse of herbicides, which are often broad-spectrum herbicides with wider application windows, has increased selection pressure on this trait in weed, which has resulted in HT weeds [157]. An increase of up to 15-fold in glyphosate herbicide use in cultivation compared to 1974 [166] was recorded, contributed by the rapid spread of RR crops globally [156,160]. Overreliance on a few HT crops and single active herbicidal ingredients has caused leaking of HT traits [160,167,168,169], with cases relating to all of the introduced HT systems being reported worldwide [170].

Recently, negative effects on human and animals from glyphosate exposure in the field and through the food chain have caused restrictions or bans on its applications in Austria, France, Germany, and Vietnam [156]. Therefore, other alternatives for glyphosate applications in agriculture, including weed management, are required for the development of sustainable agriculture with reduced reliance on one active ingredient as glyphosate [169,171]. Due to the lack of novel broad-spectrum herbicides, newly developed HT crops still rely on glyphosate and glufosinate, along with other common types of herbicides such as ALS inhibitors, synthetic auxins, HPPD inhibitors, and ACCase inhibitors [160]. To date, combination or stacking HT traits in one variety is the choice of seed companies, starting with the release of RT technology producing hybrids (Hyola^®^ 525RT, Bayer^®^ 3000TR) with glyphosate (RR) and triazine (TT) tolerance, and followed by “stacked” varieties with Triazine tolerance and Clearfield^®^ (TT+CL), which are tolerant to both triazine and imidazolinone (IMI) herbicides [172]. Strict control of the use of HT varieties and combining other weed management practices is an efficient way to manage herbicide tolerance of weeds. 

## 4. Prospects and Future Directions

**Figure 2:**
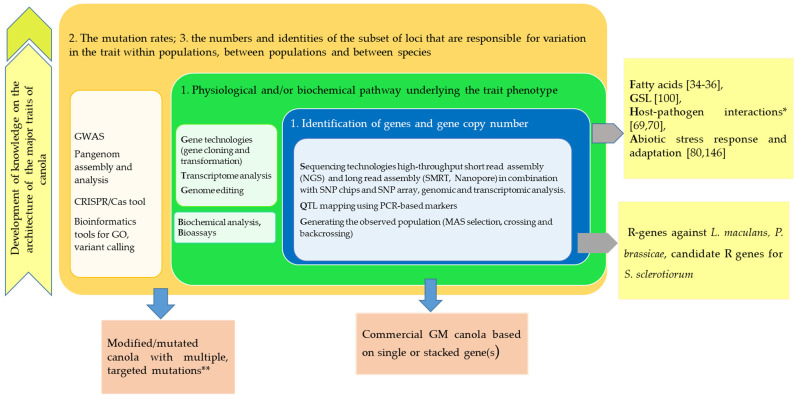
Contributions of the modern genetic and gene technologies to the understanding of the genetic architecture of the major traits of Brassica oilseed crop, adapted from Mackay [179] suggestions, and achievements in canola variety research and development. GWAS, Genome-Wide Association Study; CRISPR/Cas, the clustered regularly interspaced short palindromic repeat; GO, gene ontology; NGS, next-generation sequencing technique; SMRT, single molecule real time; SNP, single nucleotide polymorphism; QTL, quantitative trait loci; MAS, marker-assisted selection; GSL, glucosinolate; GM, genetically modified; (*) molecular biology fundamentals of these mechanisms is being established; (**) products from experimental studies.

Although conventional breeding methods are an integral part of crop improvement, modern molecular genetics and gene technologies have accelerated progress and enabled the incorporation of genetic resources across species and genera in canola. These technologies, especially gene editing, allow more detailed observations on functions of the genome compartments, as well as major genetic factors controlling biological and biochemical processes in canola, from which the obtained knowledge facilitates effective manipulation of the genetic resources towards enhancement of canola varieties. Contributions of modern genetic and gene technologies towards shaping current canola varieties are summarized in Figure 2.

As the current major market for canola crops are food, feed, and biofuel industries [17], the improvement targets prioritize oil yield and quality [9,36], and further reduction of GSL content and other antinutritional compounds [104], while simultaneously enhancing resistance against the major pathogens and tolerance to abiotic stress and herbicides [31,69,116,117,132,134,148,173,174,175]. The approval for commercial release of DHA canola [86,87] is the most recent achievement in canola improvement and exploits natural resources in fatty acid biosynthesis, where modern genetic and gene technologies play a crucial role in the success. Glucosinolates, which have been long time avoided in canola meal, are now found useful for diverse bioactivities which facilitate plant defense against non-adapted pathogen and insect pests [132,176], cancer treatment [177], and nutritional quality of canola meal [104]. Knowledge on GSL biosynthesis and their roles in physiological and biological processes of *B. napus* are being made more specific by genomic and transcriptomic approaches [100,177]. Improvement of pathogen resistance or herbicide tolerance need continued attention as pathogens and weeds can overcome these resistances after introduction of the pathogen resistant or herbicide tolerant varieties, with the pace depending on the management and rotation of those varieties in combination with other management practices [156,158,171]. The application of genomics and gene technologies in *Brassica* species have validated hypotheses and incorporated new knowledge on the evolution and the genetic diversity of *B. napus*, and explored the genetic layout of the complex genome of amphidiploid canola [63]. Genomic studies in rapeseed which used to be challenged by the complexity of the genome, are now facilitated by “omic” and gene technologies, enabling more insights about factors controlling 1,trait development and further enhancement of ca [80,100,107,134].

Facing climate change, rapid evolution of phyto-pathogens and weeds and an increasing world population, canola is assumed as an economical effective “multiple-purposes” crop fitting a broad range of markets, industries, and sustainable development policies [36]. The choice of technological approach for developing and enhancing canola varieties, such as GM canola, is affected by local legislation, research investment, and improvement targets. Although current GM canola varieties benefit in enhancement values of canola products and reducing labor costs (HT canola), the transgenes might affect performance of other genomic compartments Therefore, eliminating unessential transgenes from GM canola, environmental impact assessment and obtaining legislative approvals for commercial release of these varieties are requirements to developing a GM variety of which procedure prolongs the time reaching their markets. The world’s largest canola importer, EU, has imposed strict regulations on GM organisms, which affects technological choices in improving canola. Taking environmental and legislation perspectives, gene editing tools, such as CRISPR/Cas, are the most promising technology for enhancement of canola for commercial purpose [178]. With the advance of genomics and pan-genomics, the genetic architecture underlying response to the major pathogens and abiotic stresses such as salinity and drought need to be further dissected. Cytoplasmic genomes are attracting more interest as the new source for maximizing oil content in canola [90]. Considering factors shaping modern canola varieties, genetic and gene editing technologies are proven powerful tools for achieving new breeding targets, allowing thorough exploration and exploitation of *B. napus* genes which are largely unknown.
